# Apocynum Cannabinum in Mitral Disease

**Published:** 1905-10-07

**Authors:** 


[1] Lancet, September 30, 1905.

### APOCYNUM CANNABINUM IN MITRAL DISEASE.

Mr. A. J. B. Duprey[1] relates a case of mitral incompetency in which very striking results were obtained by the administration of tincture of apocynum cannabinum. The patient was a man of 49 years. Hepatic congestion and ascites were prominent symptoms of the case, and a mixture of digitalis with iron failed to give relief. Paracentesis was performed on many occasions, usually at an interval of six weeks or so, and enormous quantities of fluid were removed. After the new drug was added to the prescription the quantity of urine promptly increased, and the patient was soon able to leave the hospital. He did not return for a period of six months, when paracentesis was again found to be necessary. In reference to the dose of the tincture, it was noted that when this was increased to ten minims thrice daily the quantity of urine seriously diminished, and albumen, which had previously been absent, appeared. Nausea, headache, despondency, and violent epigastric pulsation were also noted. These were judged to be due to an excess of the drug; the dose was therefore reduced to six minims, and with the happiest results. One of the effects noted whilst apocynum was being taken was irritation and itching of the skin, and it is suggested that this indicates excretion of the drug through the cutaneous surface.

